# Unfolding the band structure of non-crystalline photonic band gap materials

**DOI:** 10.1038/srep13301

**Published:** 2015-08-20

**Authors:** Samuel Tsitrin, Eric Paul Williamson, Timothy Amoah, Geev Nahal, Ho Leung Chan, Marian Florescu, Weining Man

**Affiliations:** 1 San Francisco State University, San Francisco, CA, 94132 USA; 2 Advanced Technology Institute, Faculty of Engineering and Physical Sciences, University of Surrey, Guildford, Surrey, GU2 7XH, UK

## Abstract

Non-crystalline photonic band gap (PBG) materials have received increasing attention, and sizeable PBGs have been reported in quasi-crystalline structures and, more recently, in disordered structures. Band structure calculations for periodic structures produce accurate dispersion relations, which determine group velocities, dispersion, density of states and iso-frequency surfaces, and are used to predict a wide-range of optical phenomena including light propagation, excited-state decay rates, temporal broadening or compression of ultrashort pulses and complex refraction phenomena. However, band calculations for non-periodic structures employ large super-cells of hundreds to thousands building blocks, and provide little useful information other than the PBG central frequency and width. Using stereolithography, we construct cm-scale disordered PBG materials and perform microwave transmission measurements, as well as finite-difference time-domain (FDTD) simulations. The photonic dispersion relations are reconstructed from the measured and simulated phase data. Our results demonstrate the existence of sizeable PBGs in these disordered structures and provide detailed information of the effective band diagrams, dispersion relation, iso-frequency contours, and their angular dependence. Slow light phenomena are also observed in these structures near gap frequencies. This study introduces a powerful tool to investigate photonic properties of non-crystalline structures and provides important effective dispersion information, otherwise difficult to obtain.

Since the discovery of PBGs in quasi-crystalline structures^1^,^2^,^3^,^4^,^5^,^6^ and disordered media^7^,^8^,^9^,^10^,^11^,^12^, non-crystalline PBG materials have received increasing attention. Disordered photonics materials are of particular interests, due to their broadband and wide-angle properties^13^. Particularly, numerical simulations predicted that a new class of “designer” hyperuniform disordered (HUD) dielectric structures consisting of dielectric cylinders connected by dielectric wall-networks, possess sizable complete PBGs for all polarizations^7^. This prediction was later verified experimentally^8^. Even at an index-contrast as low as 1.6 vs. 1, a single-polarization PBG in a 2D HUD structure was demonstrated experimentally^9^. The intrinsic isotropy of HUD photonic structures was also proven to offer unprecedented flexibility and freedom in functional defect architecture design not limited by crystalline symmetries^8^.

For periodic structures, i.e. photonic crystals, band structure calculations produce accurate dispersion relations^14^,^15^. The photonic dispersion and the associated mode structure are then employed to determine the fundamental properties of light in periodically structured media: the local density of states (LDOS), which determines the rates of spontaneous emission^16^; the group velocities, which govern the radiation transport properties; the group velocity dispersion (GVD), which is highly relevant for evaluating the effect of nonlinearities in photonic-crystal fibers^17^; the iso-frequency surfaces, which determine the behavior of the radiation at photonic-crystal interfaces^18^; and the curvature of the dispersion relation which determines the directional properties of the propagating radiation^19^. However, band calculations for non-periodic (quasiperiodic, disordered or random) structures employ super-cells with various sizes^6^,^7^,^8^. To achieve convergence, such simulations require large supercells containing hundreds to thousands of building blocks. For a supercell encompassing *N* building blocks, the corresponding first Brillouin zone shrinks its size *N*-times, and each band is “folded” into *N* effective bands. The super-cell’s first Brillouin zone has very small spatial extent in the wave-vector space, and the resulting band structure contains a very large number of almost horizontal bands that seem to have no resemblance to the band structure of related periodic structures^9^. The only useful information in the folded band structure is the position and width of eventual band gaps and the “density” of the horizontal bands in the frequency space, which provides a visual measure of the density of states (DOS). All information regarding group velocity, group velocity dispersion, and iso-frequency contours are hidden in the flat-appearing bands and the corresponding optical mode distributions. For example, as shown in Fig. 1a (similar to Fig. 4c in Ref. 9), the large number of calculated bands near the bandgap appear to be perfectly flat and occupy a large continuous frequency range. Moreover, various isotropy metrics introduced previously are misleading, since for the folded band structure associated with a supercell they account only for changes taking place on extremely small wave-vector ranges^4^,^6^ and provide little information about the true isotropy of the dispersion relation (an artificial supercell of perfectly anisotropic periodic photonic structure will produce a similar degree of flatness in the folded band structure). A different method is needed to reveal the dispersion relations inside non-crystalline photonic structures. Experimentally measuring the phase delay of microwave radiation through cm-scale periodic photonic structures can permit the structure’s band diagrams (dispersion relations) to be reconstructed^20^. In this study we apply similar phase analysis method to our disordered system to reconstruct its “unfolded” effective band structure.

In this study, we perform both microwave experiments and detailed numerical simulations to measure the phase delay of radiation propagating through the HUD materials. By processing the phase delay data, we construct the effective band diagrams, defined by the average dispersion relations of the effective photonic modes inside the disordered structures. To validate our approach, we perform the same experimental and numerical phase analysis on a similar square lattice photonic crystal and show that the results are in good agreement with theoretical predictions. We also demonstrate that disordered PBG materials have a truly isotropic effective Brillouin zone and verify the existence of their sizeable PBGs. We ascertain the effective dispersion relation, group velocity vector, and angular dependence of HUD structures by constructing band diagrams and iso-frequency contour plots from the phase analysis.

## Methods

The design principles of the HUD PBG structures can be found in Ref. [7,^9^]. In this study, we have focused on HUD photonic materials with a 2D wall-network architecture for transverse electric (TE) polarization PBGs. A centroidal tessellation protocol is applied to a hyperuniform point pattern to generate a dual lattice. The lattice vertex pairs are connected by dielectric walls of fixed width to generate a trihedral-wall-network photonic architecture^7^,^9^. Similar design principles can be applied to obtain transverse magnetic (TM) polarization PBGs^7^,^21^ or complete PBGs^7^,^8^ and can be extended to 3D structures as well^22^.

**Figure 1:**
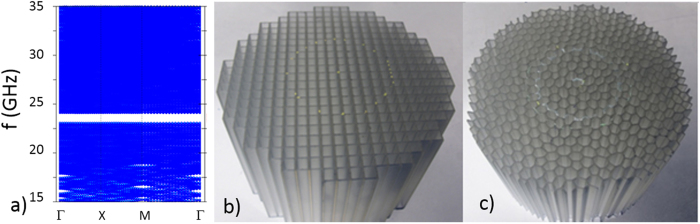
(**a**) Simulated band structure (blue) of a HUD dielectric wall-network strucutre with refractive-index contrast 1.6 vs.1. (**b**) Photo of the square lattice crystal (with lattice spacing a = 6.60 mm) (**c**) Photo of the HUD structure (average inter-vertex spacing a = 5.72 mm). The volume-filling fraction is 40.5% and the height is 100 mm for both samples.

Experimentally, we use the HUD sample and square lattice sample shown in Fig. 1b,c. The samples were fabricated with a stereolithography machine (model SLA-7000 from 3D Systems®) that produces a solid plastic model by ultraviolet laser photo-polymerization. The resin used was Accura® 60 (a clear, polycarbonate-like plastic) from 3D® Systems Corporation. Its refractive index at our experimental frequency range of 15–35 GHz is measured to be 1.6. The spatial resolution is 0.1 mm in both lateral and vertical directions. The centimeter-scale photonic structures are designed to have a nearly circular boundary with a diameter of 135 mm, which is about 23 times of the average “lattice” spacing, *a* (see Fig. 1).

Measurements were made using microwave radiation in the frequency range of 15–35 GHz (0.33 to 0.77 c/*a*), with a setup similar to the one described in Ref. 23. A single polarization mode was coupled through two horizontal facing rectangular microwave horn antennas connected to a HP8510C vector network analyzer (VNA). The horns were placed apart at a distance of 40*a* to produce approximately plane wavefronts in the center region between the two horns. The VNA is calibrated by recording the signal through free air in between the two horns. Then each sample was placed at the center between the horns with its vertical axes aligned to be perpendicular to the incident beam. Under such calibration, the VNA measured transmission is defined as the ratio between detected intensities with and without the sample in place, and the measured phase is the phase difference between the detected phase with and without the sample. To fully map the angular dependence of the transmission, we rotated each sample about its vertical axis and recorded data after every two degree rotation.

In the simulation, we used the 2D FDTD method. A 15*a* × 15*a* domain with a uniform mesh of 0.032*a* step size was defined and perfectly matched layers (PML) boundary conditions were applied. A plane wave source was placed at the left domain edge and the transmission was monitored on the right domain edge. The photonic template, either square lattice or HUD structure, is made to cover the whole domain such that that any acquired phase is purely due to internal propagation. The dielectric constant is set to *ε* = 2.56 and the wall thickness is set to *w* = 0.229*a*. A plane wave pulse with center frequency *f* = 0.5(*c*/*a*) and frequency width Δ*f* = 0.58(*c/a*) is applied, and the fields are recorded at the right edge of the sample with 1500 frequency-point resolution.

## Results and Discussions

**Figure 2:**
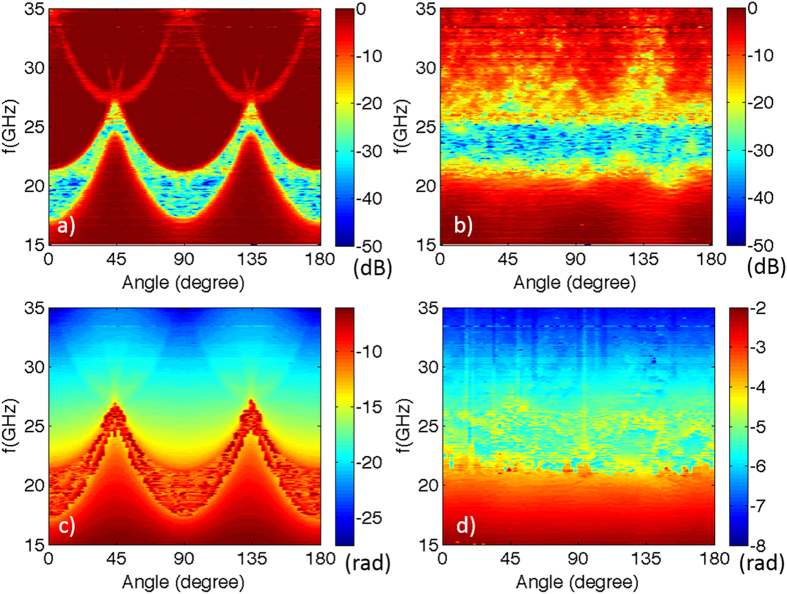
Contour plots of the experimentally measured transmission (a,b) and phase delay (c,d) as a function of frequency and incident angle for the square-lattice crystal (a,c) and HUD structure (b,d). In the square lattice, Bragg scattering is responsible for forming stop bands (blocking regions), and their center frequency and width vary rapidly with the incident angle. In the HUD sample, a truly isotropic PBG is observed around 23.5 GHz despite the low index-contrast of 1.6:1 and the lack of Bragg scattering. The measured phase delay varies with frequency continuously for propagating modes outside of stop bands and appears to be random inside the stop bands where transmission is extremely low.

In Fig. 2a,b, we use 3D color contour plots to present the measured transmission for the square lattice and the HUD sample as a function of frequency and incident angle^9^. The green-to-blue regions correspond to the stop bands. For the square lattice, the variation of the frequency range of the stop bands with incident angle prevents the formation of a PBG (blocking propagation in all directions). The HUD structure forms a truly isotropic PBG indicated by two-order-of-magnitude transmission drop around 23 GHz^9^. In Fig. 2c,d, we use 3D color contour plots to present the measured phase for the square lattice and the HUD sample as a function of frequency and incident angle. At frequencies outside of the stop bands, the measured phase varies continuously as a function of frequencies at each angle, while inside the stop bands, the measured phase acquires a random noise characteristic when the transmitted intensity is close to zero. The square lattice phase data plot shows the angular dependence in perfect agreement with the transmission intensity data. The angular-dependent dispersion relation in the square lattice can be reconstructed from the measured phase data. At frequencies close to the stop bands, the measured phase varies more quickly as a function of the frequencies, indicating a slower group velocity than that at frequencies far from the stop bands. For the HUD sample, the measured phase data is isotropic, varies continuously as a function of frequency outside of the band gap, and also presents random noise inside the stop bands.

**Figure 3:**
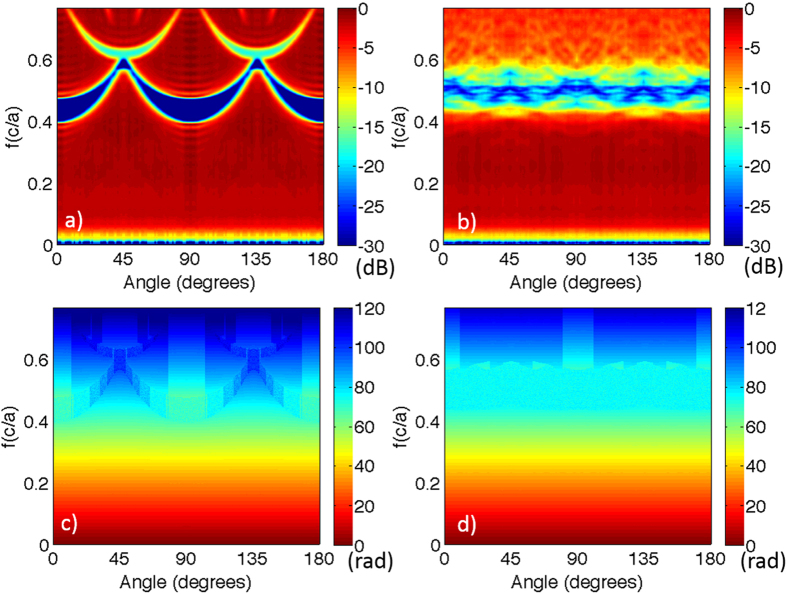
Simulated transmission and accumulated actual phase in the square lattice (a,c) and HUD (b,d) as function of frequency and incident angle for the structures analyzed in Fig. 2.

Figure 3a,b show the calculated color contour plots of the transmission, and Fig. 3c,d show the calculated color contour plots of the accumulated phase as a function of frequency and incident angle for the square lattice and the HUD structure, respectively. These simulation results mirror the experimentally obtained data presented in Fig. 2a–d. The measured data presented in Fig. 2 covers the frequency range from 15 to 35 GHz, which corresponds to the frequency range of 0.33 to 0.77 (*c*/*a*) in Fig. 3. A small number of artifacts are still present in Figs 2 and 3, caused by errors in tracking phase jumps of 2π at the lower and upper band edges where phase data becomes increasingly random.

**Figure 4:**
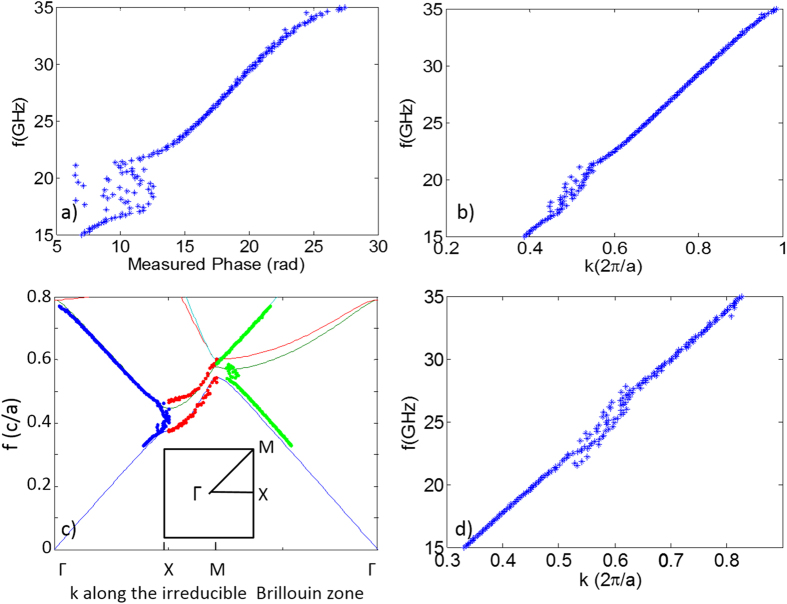
Dispersion relation reconstructed from measured phase data. (**a**) A sample plot of frequency vs. measured phase delay through the square lattice along the X direction (with 0° incident angle). (**b**) A sample plot of frequency vs. calculated wavenumber, for the square lattice along the X direction. (**c**) Dispersion relation along the irreducible Brillouin zone boundary reconstructed from measured phase data for the square lattice, plotted on top of the theoretically calculated bands results (thin solid curves). (Blue dots—the X direction, folded from data in Fig. 4b; Green dots—the M direction; and red dots—angles in between X and M). (**d**) Dispersion relation along an arbitrary direction in the HUD sample reconstructed from measured phase data.

Next, we construct the band diagram from the experimentally measured phase data. As mentioned above, the measured phase, ϕ_*m*_. is the phase difference between the detected phase delay with and without the sample in place. For example, Fig. 4a plots frequency vs. the measured phase at 0° incident angle for the square lattice. Adding the phase, 2π*fL*/*c*, accumulated in air through the known sample length *L* with measured ϕ_*m*_ in each direction gives the actual phase delay in the sample, which can be directly converted into wavenumber |*k*| of the propagating mode inside the sample along each incident angle, as shown in Fig. 4b. Contour plots for the resulting wavenumber dependence on frequency and angle in color, for both the square lattice and the HUD sample, are presented in the Supplementary Fig. S1. Figure 4c shows the reconstructed band diagram (along the irreducible first Brillouin zone boundary) for the square lattice sample using the measured wavenumber |*k*| as a function of frequency along special symmetry directions of Γ-X (0° incidence, blue dots) and Γ-M (45° incidence, green dots), as well as the measured wavenumber and frequency at the edge of the stop bands between 0° to 45° (red dots). The results agree very well with the calculated band diagram (solid curves in Fig. 4c) for the same square lattice, hence validating our method of constructing effective band diagrams using microwave phase delay measurements through finite cm-scale samples. Applying the same method to the HUD sample data yields the results shown in Fig. 4d, a plot of frequency vs. the effective measured wavenumber |*k*|. As expected, the effective dispersion relation for HUD sample is statistically isotropic, with little difference between the band structures along various directions. The group velocity (the slope of the frequency vs. wavenumber shown in Fig. 4c,d) is constant for modes far away from the bandgap, indicating radiation propagation with a well-defined energy transport velocity. In the spectral regions close to the photonic band gap, the slope of the measured dispersion become noticeably smaller, indicating a combination between slow light effects and diffusive modes near the gap edges. It is interesting to notice that despite the unavoidable scattering inside the disordered system, the effective dispersion curve is in general smooth outside of the bandgap, suggesting a meaningful definition of group velocity. Actually, in disordered systems, it is often difficult or even impossible to define a group velocity. However, the HUD system is not random, but disordered with some very strong correlations due to the high degree of hyperuniformity, hence the strongly-correlated scattering still allows a meaningful average effective group velocity to be found.

**Figure 5:**
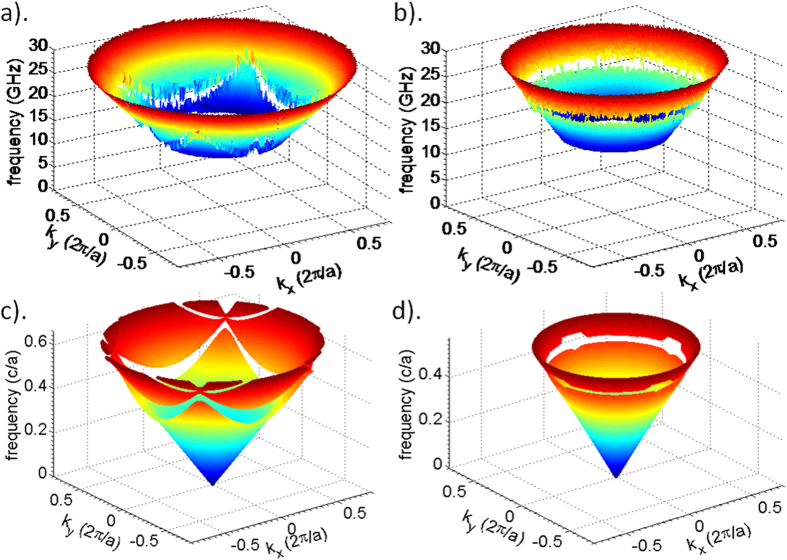
Photonic bands diagrams (dispersion relations) for (**a**) the square lattice sample reconstructed from experimentally measured phase data, (**b**) the HUD sample reconstructed based on the experimentally measured phase data, (**c**) the square lattice directly computed by solving the definite-frequency eigenstates (harmonic modes) of Maxwell’s equations14, (**d**) the HUD sample reconstructed from numerically simulated phase data.

In order to visualize the dispersion relations in all directions, we use the reconstructed wavenumbers as a function of incident angle and frequency to extract the frequency as a function of wave vector component *k*_x_ and *k*_y_ in two perpendicular directions in the incidence plane. Figure 5a,b show the band diagram constructed using the experimental phase data, while Fig. 5c shows the band diagram for the square lattice sample calculated theoretically. As previously discussed, direct band calculations lead to folded band diagrams and cannot be used to construct dispersion relations for non-periodic systems. Consequently, we use the phase information to reconstruct the effective dispersion relation for the disordered structures. Figure 5d shows the band diagram constructed using our numerically simulated phase data. To enhance the visualization of stop bands in these 3D surface plots, the random phase jumps detected inside those stop bands were eliminated in Fig. 5a,b,d. Again, the bands diagram of the square lattice constructed from the experimentally measured phase data (Fig. 5a) agrees very well with the theoretically calculated bands diagram (Fig. 5c), further validating our method of constructing effective band diagrams using phase analysis. In homogeneous dielectrics, the dispersion relations present an isotropic light cone, and frequency is proportional to wave-number. For photonic crystals, as shown in Fig. 5a,c, at long wavelengths, there is little departure from the conic dispersion relation of a homogeneous medium. Close to the Brillouin zone boundary, stop bands form at continuously changing frequencies and the band diagram distorts differently in all directions. For a crystal, the dispersion and group velocity (the slope of the frequency vs. wavenumber) have strong angular dependence. In contrast, the HUD sample shown in Fig. 5b,d presents a bandgap and a truly isotropic dispersion relation. Note that a small number of artifacts are present in simulated phase data caused by the simulation geometry and errors in tracking phase jumps of 2π at the lower and upper band edges, which create a small amount of artificial gap widening in 4 directions.

**Figure 6:**
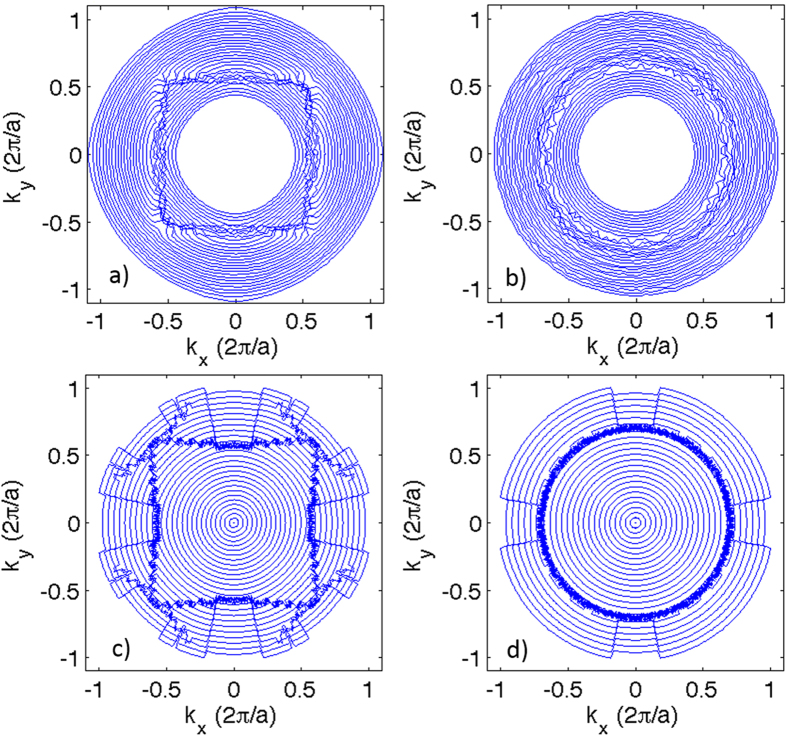
Iso-frequency contour plots. The left panel (**a,c**) displays the square lattice results and the right column displays the hyperuniform results (**b,d**). The top row (**a,b**) shows the iso-frequency contours reconstructed from measured data and the bottom row (**c,d**), the ones reconstructed from field transmission simulations.

Figure 6a,b show the iso-frequency contour plots based on our experimental data, while Fig. 6c,d present the simulated iso-frequency contour plots. Since the gradient of the frequency with respect to the wavevector represents the group velocity, iso-frequency contour plots provides direct information about the orientation of group velocity vectors in these samples. The four-fold rotational symmetry of the square lattice is clearly demonstrated by the measured and simulated iso-frequency plots. The iso-frequency contour curves deform and bend near the Brillouin zone boundaries, and the resulting angular dependent dispersion relation and group velocities provide a clear signature of the underlying structure symmetry. The first and second Brillouin zone boundaries are clearly visible due to the randomly noisy phase data inside the stop bands caused by Bragg scattering for photonic crystals at Brillouin zone boundaries. For the HUD sample, as expected, all the iso-frequency lines remain concentric circles, due to the intrinsic isotropy of the structure. The band gap (associated with the randomly noisy data) in the HUD structure is isotropic and highlights an almost circular effective “Brillouin zone”. From the above data, the group velocity vector (dispersion) as a function of incident angles and frequencies can also be found. The measured effective group velocity is obviously reduced at frequencies near stop bands (Brillouin zone boundaries) for both the square lattice sample and the HUD sample.

## Conclusions

In this study, we have used measured and simulated transmission and phase delay information to reconstruct the dispersion relations of propagating electromagnetic modes in HUD photonic materials, a new class of non-crystalline PBG structures. This methodology overcomes major issues associated with employing large supercells to generate folded band diagrams from direct band structure calculations. We have validated our approach by reconstructing the band diagrams for photonic crystals, and our experimental results agree very well with both direct band structure calculations and numerical reconstruction of dispersion relations. By comparing the iso-frequency contour plots for periodic and disordered samples, we are able to visualize the effective Brillouin zones and extract information about the group velocity fields. Our experimental and simulation results not only verify the existence of sizeable PBGs in these structures, but also provide detailed information of the effective band diagrams, dispersion relation, group velocity, and iso-frequency contours. Our results confirm the expected angular dependence of photonic crystals, and provide the first quantitative measure of the isotropic dispersion characterizing HUD photonic structures. Slow light phenomena are also observed in these structures near gap frequencies. This study presents a powerful tool to investigate photonic properties of non-crystalline structures and provides important dispersion information, which is otherwise difficult to obtain.

## Additional Information

**How to cite this article**: Tsitrin, S. *et al.* Unfolding the band structure of non-crystalline photonic band gap materials. *Sci. Rep.* **5**, 13301; doi: 10.1038/srep13301 (2015).

## Supplementary Material

Supplementary Information
