# Supplementary material for: Unfolding the band structure of non-crystalline photonic band gap materials
Source: Sci Rep. 2015 Aug 20;5:13301. doi: 10.1038/srep13301 (PMC4542607; doi:10.1038/srep13301)
Supplement: Supplementary Information [file srep13301-s1.pdf]

Unfolding the band structure of non-crystalline photonic band gap materials

[a]Sam Tsitrin, [a]Eric Williamson, [b]Timothy Amoah, [a]Geev Nahal, [a]Ho Chan, [b]Marian Florescu*, [a]Weining Man*

[a]San Francisco State University, San Francisco, CA, 94132 USA;

[b]Advanced Technology Institute, Faculty of Engineering and Physical Sciences, University of Surrey, Guildford, Surrey, GU2 7XH, UK;

*Corresponding authors: Weining Man (weining@sfsu.edu) and Marian Florescu (m.florescu@surrey.ac.uk)

Supplementary Figure S1:

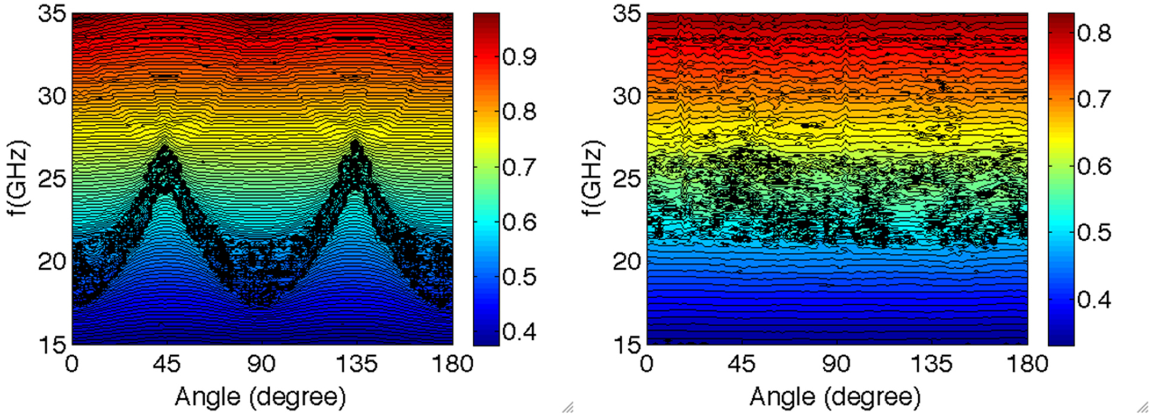

S1. Contour plots of wave number $|k|$ in the unit of $(2\pi/a)$ as a function of frequency and incident angle, obtained from the experimental phase data, for the square-lattice crystal (left) and the HUD structure (right).
